# Autoantibodies to dsDNA in the diagnosis, classification and follow-up of patients with systemic lupus erythematosus

**DOI:** 10.1016/j.jtauto.2023.100191

**Published:** 2023-01-21

**Authors:** Jan Damoiseaux, Joyce van Beers

**Affiliations:** Central Diagnostic Laboratory, Maastricht University Medical Center, Maastricht, the Netherlands

**Keywords:** Systemic lupus erythematosus, anti-dsDNA, Biomarker, Lupus nephritis

## Abstract

Autoantibodies, in particular anti-dsDNA antibodies, are increasingly used for diagnosis, classification and follow-up of patients with SLE. Since standardization of autoantibody assays is a major challenge, more attention should be paid to harmonization initiatives and better definition of required test characteristics in classification criteria. For diagnosis and follow-up separate multi-center studies are required to establish test characteristics of distinct immuno-assays for both purposes. Finally, such studies should consider not to evaluate SLE as a single disease, but as a disease with distinct subtypes.

## 1 Introduction

Systemic lupus erythematosus (SLE) is widely considered an autoimmune disease that can affect many distinct organs and tissues, and involves multiple components of both the innate and adaptive immune system. B-cells play an important role in the immunopathogenesis as reflected by the common manifestation of hypergammaglobulinemia and the treatment with B-cell targeting drugs. Within the elevated IgG levels a huge variety of autoantibodies has been described [1], only some of which have been introduced in clinical practice. These autoantibodies are increasingly incorporated in the successive classification criteria for SLE [2]. In particular anti-dsDNA antibodies are considered fundamental in the diagnostic work-up of an SLE patient and may also be useful for follow-up [3]. Over the years, many novel technologies for detection of anti-dsDNA antibodies have entered the routine clinical laboratories resulting in a wide heterogeneity in test results [4,5]. Since most results are expressed in international units, suggesting that assays are well standardized, the heterogeneity in assays and test results is often overlooked. Here we provide a short historical overview of autoantibodies in the classification criteria of SLE with a focus on anti-dsDNA antibodies, we discuss the impact of considering SLE as a single disease *versus* distinguishing SLE subtypes, and the limitations in using anti-dsDNA antibodies as a marker for existing, or even upcoming disease activity.

## 2 Historical perspective

Disease criteria are generally based on clinical manifestations, pathological evaluations and laboratory results. Diagnostic criteria will enable the clinician to establish the most likely diagnosis, while classification criteria are more stringent in order to only include patients with a definite diagnosis in studies, either for unravelling the immunopathogenesis or to evaluate the efficacy of new drugs. Over the last decades no diagnostic criteria for SLE have been published and/or endorsed by the rheumatology societies. Searches on the internet for diagnostic criteria most often erroneously refer back to the existing classification criteria.

The first classification criteria for SLE were composed by the American Rheumatism Association (ARA) in 1971 [6]. They included two laboratory tests that rather indirectly detected the presence of autoantibodies: the lupus erythematosus (LE) test and the false-positive venereal disease research laboratory (VDLR) test. The first is based on phagocytosed nuclear material as observed in blood smears, while the second is a flocculation test induced by antiphospholipid antibodies. This latter test was originally designed to detect infection with *Treponema pallidum*, but subsequently appeared false-positive in patient with lupus. The 1982 revised classification criteria introduced anti-nuclear antibodies (ANA) as a separate criterion and added anti-dsDNA and anti-Sm antibodies to the criterion for autoantibodies with defined antigen-specificity, next to the LE and false-positive VDRL test [7]. Revision of these criteria in 1997, released by the American College of Rheumatology (ACR, previously ARA), resulted in removal of the LE test and addition of anti-phospholipid antibodies, i.e., lupus anticoagulant and anti-cardiolipin antibodies (IgM and IgG) [8]. The subsequent Systemic Lupus International Collaborating Clinics (SLICCS) criteria introduced the direct Coombs test, but also added anti-β2-glycoprotein I antibodies to the category of antiphospholipid antibodies [9]. Moreover, besides IgM and IgG isotypes, also IgA antiphospholipid antibodies were included. The most recent classification criteria were established by a collaboration between the European Alliance of Associations for Rheumatology (EULAR, previously European League Against Rheumatism) and the ACR [10]. These criteria excluded the long-lasting false-positive VDRL test as well as the recently included direct Coombs test, while the ANA became an entry criterion. Importantly, the anti-dsDNA remained an essential item in the classification criteria for SLE after the first introduction in the 1982 ACR criteria.

The history of anti-dsDNA antibodies has been extensively reviewed by Mummert et al. [4]. The first clinically available assays for anti-dsDNA antibodies included the Farr assay, a liquid phase precipitation assay, and the *Crithidia luciliae* immunofluorescent test (CLIFT). The first can not distinguish between distinct immunoglobulin isotypes, but because the test is selective for high-affinity antibodies it detects preferentially IgG. The CLIFT identifies reactivity with dsDNA in the kinetoplast of the protozoan organism. Since this dsDNA is devoid of proteins, there is no interference of autoantibodies to histones or nucleosomes. Since both types of assays have drawbacks in clinical practice, i.e., the need for radioactivity and being labor intensive, respectively, these assays are increasingly replaced by automated, high-throughput analyzers that detect the anti-dsDNA antibodies by solid-phase, ELISA-like methodologies that have entered the market in the last two decades [11]. However, these assays, in general, have distinct test-characteristics in being more sensitive, but less specific.

The introduction of an internationally accepted standard preparation for anti-dsDNA antibodies as endorsed by the World Health Organization (WHO) has enabled to calibrate the distinct immuno-assays that are used in clinical practice and to express the results in international units (IU) [12]. This has enforced the misconception that results obtained by distinct assays are interchangeable [13]. It should be realized that each individual patient generates a unique set of anti-dsDNA antibodies that is different, in terms of epitope recognition, isotype, affinity, and glycosylation, from the antibodies of another patient. Although assays may be calibrated against the WHO standard in such a way that this standard reveals the same test-result in these assays, this does not prevent individual patient sera to reveal different results in assays that differ in antigen source, epitope exposition and/or salt concentration in the assay-buffers used [4]. Hence, standardization of autoantibody assays seems to be an utopia [14], and this seems to be underestimated in the classification criteria regarding anti-dsDNA antibodies [5]. Indeed, over the sequential sets of classification criteria there has been little attention for how test results are to be interpreted. Both the 1982 ARA and the 1997 ACR criteria defined that the antibodies should be directed to native DNA at an abnormal titer [7,8]. It was not specified if abnormal refers to healthy individuals or disease controls. Moreover, the term titer suggests that the assay is to be performed with multiple serum dilutions as is the case in the CLIFT. The 2012 SLICC criteria defined that the anti-dsDNA antibodies should be above the laboratory reference range. However, in case ELISA is used the level should be twice above the laboratory reference range [9]. This addition was based on the experience that, in general, ELISAs are less specific for anti-dsDNA antibodies, but this also implies that according to the SLICC consortium the respective diagnostic companies and/or clinical laboratories did not correctly define the cut-off of these assays. The most recent 2019 EULAR/ACR criteria define that the anti-dsDNA antibodies detected by immuno-assays should have ≥90% specificity for SLE against relevant disease controls [10]. Given the high impact of a positive result, *i.e.*, 6 out of 10 points required for classification, this level of specificity is rather low for a relatively rare disease as SLE. More importantly, it is not evident from available studies that even this low level of specificity is achieved by multiple commercial assays currently used in routine clinical laboratories [15].

### 2.1 Autoantibody testing in a disease with multiple faces

**Fig. 1:**
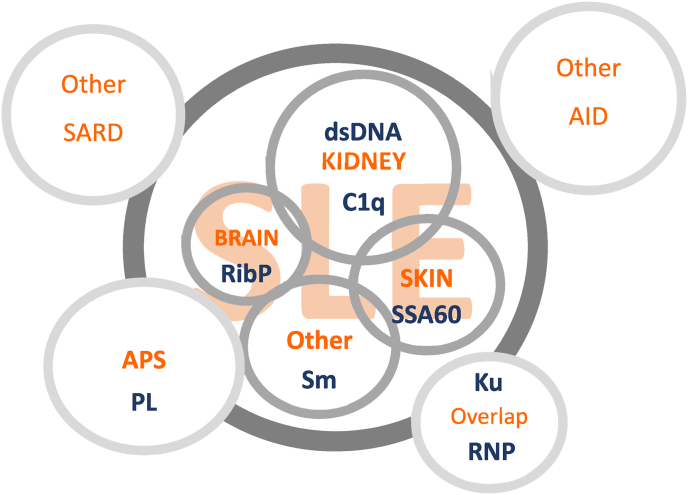
Multiple faces of SLE. This simplified concept of the distinction of different SLE subtypes illustrates the importance of considering the test characteristics of autoantibodies in the context of these subtypes. The size of the circles does not represent relative representation of the disease (sub)types. In particular in terms of classification this enables better understanding of the pathophysiology and definition of primary outcomes of clinical trials. Importantly, the overlap in clinical manifestations with other (systemic) autoimmune diseases, should be taken into account. Disease (sub)types are depicted in orange; antigens recognized by autoantibodies are depicted in blue. Abbreviations: AID, autoimmune diseases; APS, antiphospholipid syndrome; C1q, complement factor C1q; dsDNA, double stranded deoxyribonucleic acids; PL, phospholipids; RibP, ribosomal P-proteins; RNP, ribonucleoprotein; SARD, systemic autoimmune rheumatic diseases; SLE, systemic lupus erythematosus; Sm, Smith; SSA, Sjögren’s syndrome A.

What is SLE? This seems to be a simple question, but if you ask this question to clinicians from different disciplines distinct descriptions will be given. Indeed, SLE is a disease with multiple faces based on the organ that is predominantly involved, varying from for instance the kidneys, the central nervous system, or the skin. This heterogeneity within the SLE population is a challenge for the establishment of useful classification criteria, as recently discussed by Rekvig [16]. Based on the 1997 ACR criteria, requiring a minimum of 4 criteria out of a total of 11, the number of combinations (each combination defines one clinical phenotype) is 330. Rekvig stated that “*this number is not in conformity with, and argues against the statement that the unremitting refinements of SLE classification criteria define patient cohorts suitable for insightful and basic studies in SLE*”. Obviously, the number of 330 only holds if the 11 criteria are completely independent phenomena, which is evidently not the case. For instance, if a patient has anti-dsDNA antibodies this patient most often also as ANA. Nevertheless, it may be important to differentiate distinct subtypes based on clinical manifestations and associated autoantibodies (Fig. 1). There may be overlap with other subtypes, but subtypes might also be defined as the so-called overlap-syndromes including the antiphospholipid syndrome (APS) and mixed connective tissue disease (MCTD). Clinical and laboratory features might be shared with other systemic autoimmune rheumatic diseases or organ-specific autoimmune diseases. Eventually, it can even be argued that, in particular, the systemic autoimmune rheumatic diseases should be considered as a continues spectrum with many overlapping features instead of distinct disease entities.

In most autoimmune diseases sensitivity of hallmark autoantibodies does not reach 100%. For anti-dsDNA antibodies in SLE this is estimated to be about 60%. Therefore, there is an ongoing search for covering the serological gap, either by identifying additional autoantibodies, like anti-Sm, anti-RibP, or anti-SSA60 antibodies that each may define distinct SLE subtypes, or by technical improvements to increase the sensitivity of the anti-dsDNA antibodies. The latter approach has consequences for both classification as well as diagnosis. As already alluded to above, the intention of classification criteria is to define a well-defined disease population to unravel pathophysiological mechanisms and/or evaluate novel therapeutic options. Already in the sixties of the previous century a strong association between anti-dsDNA antibodies, as detected by the at that time available assays, and lupus nephritis has been established. If the sensitivity of the assays for anti-dsDNA antibodies is increased, it is very likely that this reduces the association with lupus nephritis and this may impact the understanding of the mechanisms that are pathogenic in the kidneys. Also for diagnostic purposes an increase in sensitivity may be detrimental, because an increase in sensitivity most often comes with a decrease in specificity. As illustrated in Table 1, an estimated 10% increase in sensitivity is accompanied by decrease in specificity in fluorescent-enzyme immuno-assay (FEIA) and chemiluminescent immuno-assay (CLIA) by 4.0 and 6.4%, respectively. This strongly affects the diagnostic value of a positive test result as illustrated by a 3–5x reduction in positive likelihood ratio, while the negative likelihood ratio only reveals a minor improvement. This implies that the assays with low specificity are less suitable in situations with a low pre-test probability. Inclusion of such patients with low pre-test probability is often the consequence of testing algorithms used in routine clinical laboratories that prescribe that anti-dsDNA antibody testing is to be added if ANA is positive in the diagnostic work-up of a patient. Such algorithms are generally based on guidelines and/or recommendations. These recommendations, however, are often defined in more detail than just “*if ANA is positive*”. For instance, the recommendations of the European Autoimmunity Standardization Initiative (EASI) and the International Union of Immunological Societies (IUIS) is formulated as “*If ANA result is positive, testing for anti-dsDNA antibodies is advised when there is clinical suspicion of SLE*” [17]. Since many laboratories are not informed about the clinical suspicion linked to the requested ANA, the translation of the recommendation into the testing algorithm results in many anti-dsDNA tests being performed out of the appropriate clinical context and, consequently, false-positive results when using assays with low specificity.

**Table 1:** The effect of test characteristics on the interpretation of a test result.

	Sensitivity (%)	Specificity (%)	LR+	LR-
CLIFT^a^	60^b^	98.7 (97.7–99.5)	46.2	0.41
FEIA	70^b^	94.7 (91.7–96.7)	13.2	0.32
CLIA	70^b^	92.3 (83.6–96.6)	9.1	0.33

a Abbreviations: CLIA, chemiluminescence assay; CLIFT, *Crithidia luciliae* indirect immunofluorescent test; FEIA, fluorescent-enzyme immuno-assay; LR, likelihood ratio.

b Estimated value because there is enormous variation in the sensitivities published partially due to differences in the composition of the tested SLE cohort.

## 3 Anti-dsDNA antibodies in relation to disease activity

**Fig. 2:**
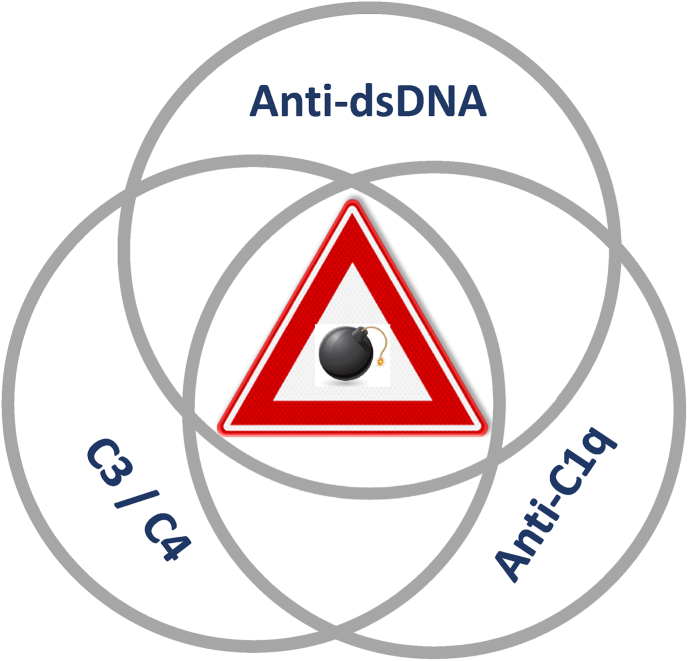
Multi-parameter prediction of relapse. Although anti-dsDNA antibodies have been associated with clinical relapse in SLE patients, this predictive value may be enhanced by taking into account anti-C1q antibodies and levels of complement factors C3 and C4. A large multi-center study is required to evaluate the distinct available assays in a prospective, clinically well-defined SLE cohort.

Especially if autoantibodies are considered to be pathogenic, it is to be expected that the level of such antibody correlates with disease activity and may even precede a clinical relapse. For anti-dsDNA antibodies in relation to lupus nephritis these antibodies either bind to dsDNA that is captured by the glomerular basement membrane (GBM) or that immune-complexes are formed in the circulation and subsequently deposit in the glomeruli [3]. Since both dsDNA as well as the GBM are negatively charged, a direct interaction is unlikely, but extracellular dsDNA may still be linked to histones in the form of nucleosomes which enables binding to the GBM [18]. Circulating immune-complexes may be deposited in the subendothelial space and mesangium, causing glomerulonephritis, or in the subepithelial space between the podocytes, causing membranous nephropathy [19]. The locally formed and/or deposited immune-complexes will activate the classical pathway of the complement system resulting in reduced circulating C3 and C4 levels. The reduction in C3 may be masked by increased production in the liver because C3 is part of the acute phase response. Interestingly, lupus nephritis is also strongly associated with anti-C1q antibodies and these autoantibodies are known to enhance immunopathology in animal models [20,21]. These data suggest that the combination of anti-dsDNA and anti-C1q antibodies together with C3 and C4 levels may have the best predictive value for disease exacerbation in SLE (Fig. 2).

Multiple studies have addressed the association between anti-dsDNA antibodies and disease activity in SLE; rises in anti-dsDNA antibodies even may herald an impending flare (reviewed in Refs. [4,22]. Most studies that refer to the predictive value of anti-dsDNA antibodies in terms of upcoming disease activity refer to Bootsma et al. [23]. In this study 18 of 34 patients experienced 26 relapses within a time frame of two years. The results showed that an increase in anti-dsDNA antibodies, as determined by a commercial Farr assay and an in-house IgG class ELISA, is significantly associated with a clinical relapse within a time frame of two years. The strength of this study is the monthly evaluations of anti-dsDNA antibody levels and the clear definition of a rise in antibody level by taking into account the type of assay. The shortcomings are the small sample size, the heterogeneity of the SLE population, and the lack of a definition for a minor or major relapse. Interestingly, only increases in anti-dsDNA antibody levels measured by Farr assay showed a highly increased odds ratio (5.4; statistically not significant) for developing renal involvement. Unfortunately, the respective commercial assay is no longer available. As also established for ANCA-associated vasculitis [24,25], predicting disease relapses by rises in autoantibody levels should take into account disease subtype, definitions of relapse and rise in autoantibody level, frequency of autoantibody measurements, and type of assay used. As for diagnostic application, also for follow-up purposes sufficiently large studies comparing different immuno-assays in the same patient cohort are lacking. For follow-up this is even more challenging than for diagnosis of SLE. Surprisingly, although solid data are lacking for clinical interpretation of rises in anti-dsDNA antibody levels as measured in distinct immuno-assays, the majority of clinicians utilizes these antibodies to monitor disease activity in patients with SLE. Moreover, this application of the assay is beyond the intention-for-use as indicated in the inserts of the currently available commercial assays and thereby officially restricts the routine clinical laboratories in using the assay for this purpose.

## 4 Summary and conclusions

In SLE a wide variety of autoantibodies has been described, but only few have a sufficiently strong association for inclusion in classification criteria [1]. In particular anti-dsDNA antibodies have consistently been part of such criteria and their contribution to the classification of SLE has increased. Indeed, the current classification criteria reward 6 points for anti-dsDNA antibodies, while 10 points are required for definite classification [10]. In the context of lacking standardization of autoantibody assays, including anti-dsDNA antibodies, surprisingly little attention is paid to the impact of assay choice and result interpretation [4,5,11]. Moreover, it has been questioned if the current classification system is appropriate for a clinically heterogeneous disease as SLE [16]. Several autoantibodies clearly associate with distinct SLE subtypes. Changes in test characteristics of such autoantibodies evidently may disturb this association.

These drawbacks also impact the diagnostic work-up of patients suspected of SLE. Since diagnostic criteria for SLE are lacking, classification criteria are often erroneously applied. Moreover, testing algorithms in most routine clinical laboratories automatically add testing for anti-dsDNA antibodies if ANA are positive, even if there is no clinical suspicion of SLE. In such overall low pre-test probability populations a test with high specificity is crucial. Altogether, there is a need for an extensive multi-center study that compares distinct available assays for anti-dsDNA antibodies in the same clinically well-defined SLE cohort. As in ANCA-associated vasculitis, such a study enables better alignment of test results based on likelihood ratio's for test result intervals [[26], [27], [28]].

With respect to monitoring disease activity, and even prediction of clinical relapses, by the level of anti-dsDNA antibodies there remain multiple hurdles to be taken. Currently available assays are not marketed for this purpose, but clinicians routinely request anti-dsDNA antibody tests for follow-up. Again, a large multi-center study is required to evaluate the distinct available assays in the same clinically well-defined SLE cohort. In such a study it is important to differentiate between SLE subtypes and clearly define what is to be considered a relapse and rise in autoantibody level. Besides anti-dsDNA antibodies, also anti-C1q antibodies and factors of the classical complement pathway should be taken into account. Preferentially, this should be a prospective study, which will be a major challenge, but which is essential to position anti-dsDNA antibody testing in monitoring SLE patients.

## Declaration of competing interest

The authors declare the following financial interests/personal relationships which may be considered as potential competing interests:Jan Damoiseaux reports a relationship with 10.13039/100011033Thermo Fisher Scientific ImmunoDiagnostics Division that includes: consulting or advisory and speaking and lecture fees. Jan Damoiseaux reports a relationship with Werfen that includes: consulting or advisory. Jan Damoiseaux reports a relationship with EUROIMMUN Medizinische Labordiagnostika AG that includes: speaking and lecture fees.

## Data Availability

No data was used for the research described in the article.
